# Evolutionary dynamics of heparan sulfate utilization by SARS-CoV-2

**DOI:** 10.1128/mbio.01303-25

**Published:** 2025-06-23

**Authors:** Shuhei Higuchi, Yafei Liu, Jun Shimizu, Chikako Ono, Yumi Itoh, Wataru Nakai, Hui Jin, Kazuki Kishida, Kazuo Takayama, Toru Okamoto, Yoshiko Murakami, Taroh Kinoshita, Yoshiharu Matsuura, Tatsuo Shioda, Hisashi Arase

**Affiliations:** 1 Center for Advanced Modalities and DDS, Osaka University 13013, Suita, Osaka, Japan; 2 Department of Immunochemistry, Research Institute for Microbial Diseases, Osaka University 13013, Suita, Osaka, Japan; 3 MiCAN Technologies Inc., Kyoto, Japan; 4 Center for Infectious Disease Education and Research, Osaka University 13013, Suita, Osaka, Japan; 5 Laboratory of Virus Control, Research Institute for Microbial Diseases, Osaka University 13013, Suita, Osaka, Japan; 6 Department of Microbiology, Juntendo University School of Medicine 73362, Bunkyo, Tokyo, Japan; 7 Laboratory of Immunochemistry, World Premier International Immunology Frontier Research Center, Osaka University 13013, Suita, Osaka, Japan; 8 Center for iPS Cell Research and Application, Kyoto University 12918, Kyoto, Japan; 9 Laboratory of Immunoglycobiology, Research Institute for Microbial Diseases, Osaka University 13013, Suita, Osaka, Japan; 10 Department of Viral Infections, Research Institute for Microbial Diseases, Osaka University 13013, Suita, Osaka, Japan; Washington University in St. Louis School of Medicine, St. Louis, Missouri, USA

**Keywords:** SARS-CoV-2, Omicron, heparan sulfate, TMPRSS2

## Abstract

Severe acute respiratory syndrome coronavirus 2 (SARS-CoV-2) Omicron variants have acquired enhanced infectivity compared to earlier variants. To elucidate the underlying molecular mechanisms, we conducted CRISPR library screening to identify cell surface molecules that interact with the Omicron spike protein. Our findings revealed a significantly higher affinity between the Omicron spike and cell surface heparan sulfate compared to the wild-type spike. This increased binding affinity enables Omicron variants to infect cells expressing low levels of ACE2, which are minimally infected by the wild-type virus. Mutational analysis of heparan sulfate binding sites on the Omicron spike protein, coupled with electrostatic potential mapping, suggested that the accumulation of positively charged mutations has contributed to the enhanced heparan sulfate binding. Comparative analysis of heparan sulfate binding among Omicron subvariants—including BA.1, BA.2, BA.4, BA.5, XBB.1, and BA.2.86—revealed that most are likely to bind efficiently to heparan sulfate, but potential heparan sulfate binding sites of the spike protein have shifted from the early Omicron variants to more recent ones. Furthermore, we discovered that cell surface heparan sulfate proteoglycans are cleaved by TMPRSS2, a protease essential for wild-type SARS-CoV-2 infection. These findings suggest that SARS-CoV-2 is evolving to enhance its infectivity by optimizing its interaction with cell surface heparan sulfate.

**IMPORTANCE:**

The Omicron variant has evolved to become highly infectious by acquiring numerous mutations. Understanding the impact of these mutations can provide valuable insights into the drivers of viral evolution and aid in the development of improved viral surveillance and vaccines. Our study demonstrates that the Omicron variants contain mutations that enhance their ability to bind to heparan sulfate. Highly infectious human viruses often utilize heparan sulfate for infection, suggesting that heparan sulfate likely plays a crucial role in viral adaptation to human hosts. Furthermore, we found that cell surface heparan sulfate proteoglycans are sensitive to TMPRSS2, while most other cell surface proteins are resistant to TMPRSS2. Given that TMPRSS2 is known to enhance the infectivity of earlier severe acute respiratory syndrome coronavirus 2 variants but cleaves heparan sulfate proteoglycans, it is probable that the high heparan sulfate binding acquired by the Omicron variant contributes to its decreased infectivity against TMPRSS2-expressing cells compared to earlier variants.

## INTRODUCTION

The severe acute respiratory syndrome coronavirus 2 (SARS-CoV-2) Omicron variant has acquired more than 30 mutations in the spike protein compared to the wild-type (WT; B lineage) SARS-CoV-2 (1). The ectodomain of the spike protein is composed of the S1 and S2 subunits, with the S1 subunit containing the receptor-binding domain (RBD) and N-terminal domain. The S1 subunit is responsible for interaction with host receptors, such as the viral entry receptor ACE2 (2, 3), neuropilin-1 (4, 5), C-type lectins (6, 7), heparan sulfate (HS) (8), and others. Thus, neutralizing antibodies (Abs) against the S1 subunit of the spike protein plays an important role in protection against SARS-CoV-2 infection (9, 10). The Omicron variant is thought to have acquired a number of mutations in the S1 subunit to evade most of the neutralizing Abs produced upon previous SARS-CoV-2 infection or vaccination with spike protein (11–13). In addition, the interaction between spike protein and host receptors also appears to be affected by the mutations in the S1 subunit. Although the effects of spike mutations on ACE2 binding have been extensively studied (14–16), their effects on the binding of cell surface molecules other than ACE2 have remained unclear.

The S2 subunit of the spike protein is responsible for membrane fusion, which requires cleavage by a host protease after ACE2 binding of the S1 subunit (17). While the S2 subunit of the WT spike protein is preferentially cleaved by the serine protease TMPRSS2 at the plasma membrane, the S2 subunit of the Omicron spike protein is mainly cleaved by the cysteine protease cathepsins in endosomes (18–20). This suggests that the spike mutations acquired by the Omicron variant are also involved in the altered protease usage by the SARS-CoV-2 virus.

Since the emergence of the original Omicron variant BA.1, the Omicron lineage has given rise to several subvariants, such as BA.1 to BA.5, the recombinant subvariant of two BA.2 sublineages, XBB, and BA.2.86, a descendant of the BA.2 subvariant. Currently, the JN.1 subvariants, a descendant of the BA.2.86 subvariant, and the JN.1 sublineages, including the KP.2, KP.3, and LB.1 subvariants, are dominant across the globe as of June 2024 (COVID-19 epidemiological update—15 July 2024; https://www.who.int/publications/m/item/covid-19-epidemiological-update-edition-169). Each Omicron subvariant exhibits a distinct capacity for immune escape, ACE2 binding, or TMPRSS2 dependence (21–23). Elucidating the characteristics of the spike protein of the Omicron subvariants is critical to understanding the mechanisms of SARS-CoV-2 evolution. In this study, we examined the impact of the spike mutations found in the Omicron variant on the interaction between the spike protein and the cell surface molecule.

## RESULTS

### The SARS-CoV-2 Omicron variant has acquired the ability to infect low-level ACE2-expressing cells

**Fig 1:**
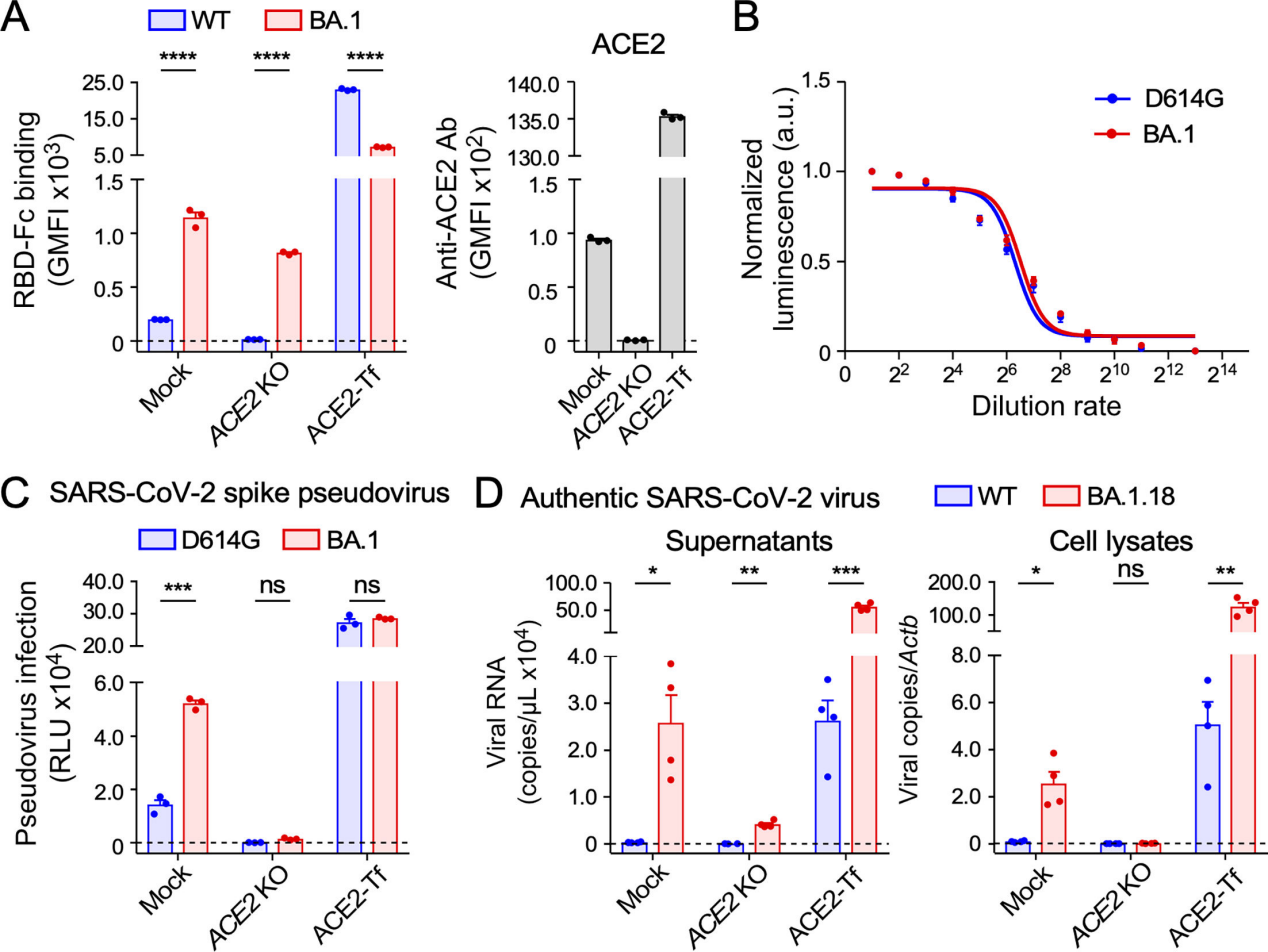
The Omicron variant infects HEK293T cells expressing low levels of endogenous ACE2. (**A**) Binding of WT (B lineage) or Omicron (BA.1) RBD fused to human IgG Fc (RBD-Fc), or anti-ACE2 Ab to mock, *ACE2* KO, or ACE2-transfected (Tf) HEK293T cells. GMFI, geometric mean fluorescence intensity. (**B**) Titration of pseudoviruses bearing the SARS-CoV-2 D614G or BA.1 spike using VSV-G-Tf HEK293T cells. (**C**) Infection of mock, *ACE2* KO, or ACE2-Tf HEK293T cells with D614G or BA.1 pseudovirus. RLU, relative luminescence units. (**D**) Infection of mock, *ACE2* KO, or ACE2-Tf HEK293T cells with authentic SARS-CoV-2 WT or Omicron (BA.1.18) variant. Viral RNA in supernatants or cell lysates at 24 hours post-inoculation (hpi) is shown. Lysate RNA was normalized to *Actb*. Data are mean ± SEM of three to four technical replicates. Statistical analysis was performed using two-way analysis of variance (ANOVA) with Sidak’s multiple comparison tests in panels **A** and **C** and unpaired two-tailed Welch’s *t*-tests between WT and BA.1.18 in panel **D**; *P < 0.05, **P < 0.01, ***P < 0.001, and ****P < 0.0001; ns, not significant. Data are representative of two to three independent experiments.

The Omicron variant has acquired a number of mutations on the surface of the RBD to escape from neutralizing Abs. This has raised the possibility that the Omicron spike protein might alter its binding specificity due to these mutations acquired by the Omicron variant. When we analyzed the specificity of RBD using recombinant RBD fused to human IgG Fc (RBD-Fc), both WT and Omicron (BA.1) RBD-Fc bound well to ACE2-transfected HEK293T cells (Fig. 1A). Interestingly, the BA.1 RBD-Fc, but not the WT RBD-Fc, bound to mock HEK293T cells expressing low levels of endogenous ACE2 as well as to *ACE2* knockout (KO) HEK293T cells (Fig. 1A). This suggests that the BA.1 variant has acquired the ability to bind to certain cell surface molecules other than ACE2.

We then analyzed the effect of BA.1 RBD binding molecules on SARS-CoV-2 infectivity using vesicular stomatitis virus (VSV)/ΔG-Luc pseudoviruses carrying the SARS-CoV-2 D614G spike (containing only the D614G mutation) or the BA.1 spike protein. Both pseudoviruses equally infected VSV-G-transfected HEK293T cells, which are infected with pseudoviruses ACE2-independently, indicating that their pseudovirus titers were almost the same (Fig. 1B). We performed a pseudovirus infection assay for HEK293T cells under different ACE2 expression levels: mock (HEK293T cells expressing low levels of ACE2), *ACE2* KO, and ACE2-transfected cells. Although both D614G and BA.1 pseudoviruses infected ACE2-transfected HEK293T cells equally, the BA.1 pseudovirus showed enhanced infectivity to mock HEK293T cells expressing low levels of ACE2 compared to the D614G pseudovirus (Fig. 1C). On the other hand, the BA.1 pseudovirus was unable to infect *ACE2* KO HEK293T cells, indicating that ACE2 is an essential receptor for SARS-CoV-2 infection, even though the BA.1 RBD binds to *ACE2* KO HEK293T cells. Consistent with the results obtained using the pseudoviruses, the infection efficiency of the authentic SARS-CoV-2 Omicron (BA.1.18) virus to mock HEK293T cells expressing low levels of ACE2 was quite higher than that of the WT SARS-CoV-2 virus (Fig. 1D). Taken together, these findings suggest that the SARS-CoV-2 Omicron variant has acquired the ability to bind to certain cell surface molecules other than ACE2, which appears to be related to its infectivity to low-level ACE2-expressing cells that are only negligibly infected by the WT SARS-CoV-2 virus.

### The Omicron variant has acquired the ability to bind to cell surface HS

**Fig 2:**
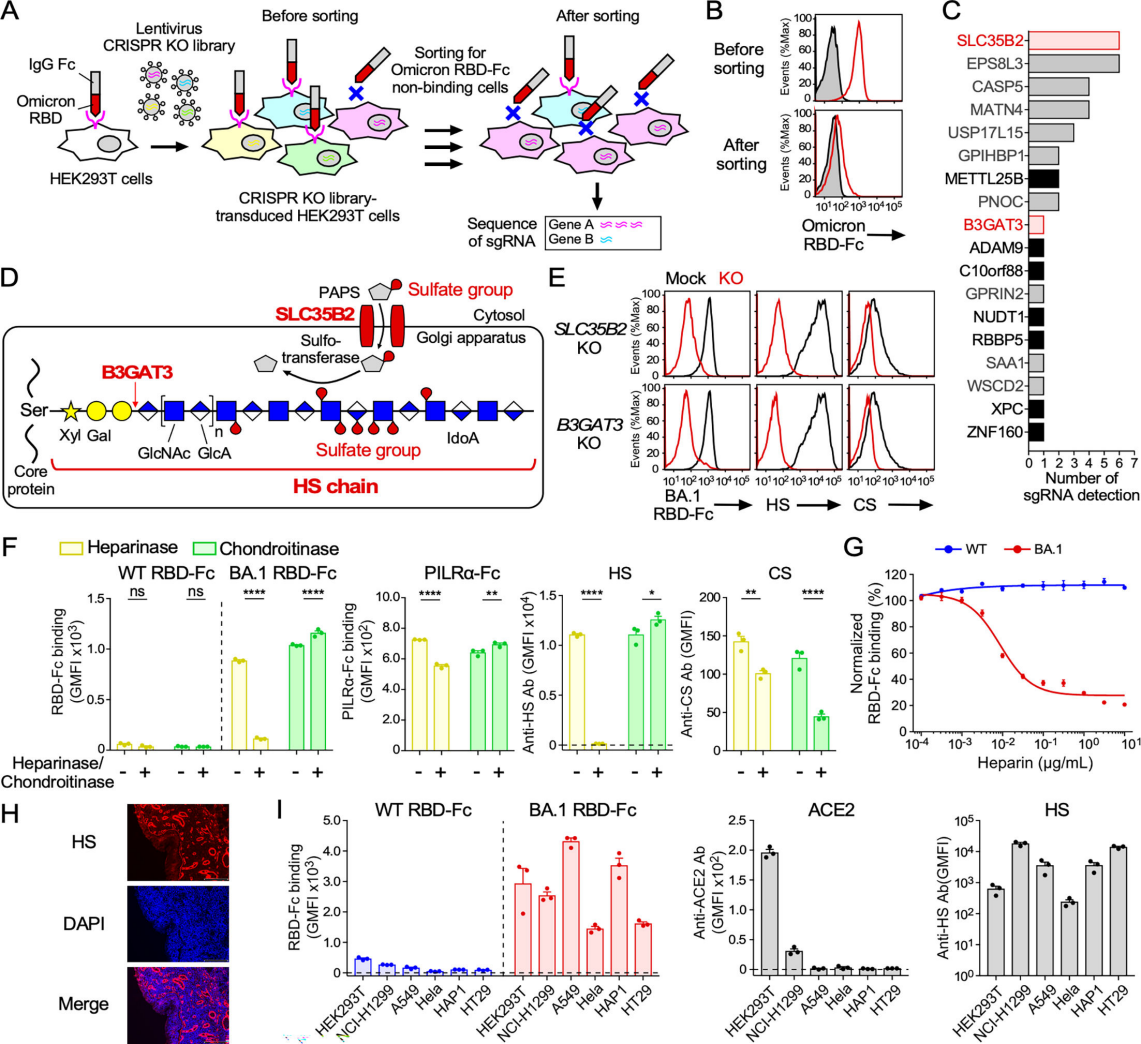
ACE2-independent binding of the Omicron RBD to cell surface HS. (**A**) CRISPR KO library screening scheme to identify Omicron RBD binding molecules expressed on HEK293T cells. (**B**) Binding of CRISPR KO library-transduced HEK293T cells with (red line) or without (shaded gray) Omicron RBD-Fc, before and after sorting. (**C**) Number of sgRNA sequences identified in Omicron RBD-Fc non-binding cells after sorting. Red: GAG-synthesis-related genes; black: other expressed genes; gray: non-expressed genes in HEK293T cells. (**D**) HS biosynthetic pathway highlighting SLC35B2 and B3GAT3. PAPS, 3'-phosphoadenosine-5'-phosphosulfate; Xyl, xylose; Gal, galactose; GlcNAc, N-acetylglucosamine; GlcA, glucuronic acid; IdoA, iduronic acid. (**E**) Binding of BA.1 RBD-Fc, anti-HS Ab, or anti-CS Ab to mock (black line), or *SLC35B2* or *B3GAT3* KO (red line) HEK293T cells. (**F**) Binding of WT or BA.1 RBD-Fc, PILRα-Fc, anti-HS Ab, or anti-CS Ab to *ACE2* KO HEK293T cells pretreated with (+) or without (–) heparinase or chondroitinase. (**G**) Binding of WT or BA.1 RBD-Fc to HEK293T cells at different heparin concentrations. RBD-Fc binding was normalized to binding in the absence of heparin. (**H**) Immunofluorescence of human nasal tissue with anti-HS Ab and 4', 6-diamidino-2-phenylindole (DAPI). Scale bar, 200 µm. (**I**) Binding of WT or BA.1 RBD-Fc, anti-ACE2 Ab, or anti-HS Ab to cell lines. Data are mean ± SEM of three to four technical replicates. Statistical analysis was performed using two-way ANOVA with Sidak’s multiple comparison tests in panel **F**; *P < 0.05, **P < 0.01, and ****P < 0.0001; ns, not significant. Data are representative of two to three independent experiments.

To identify the Omicron RBD binding molecules, we employed the CRISPR-Cas9 KO library screening system (24, 25) (Fig. 2A). Cells that had lost the ability to bind to Omicron RBD-Fc were enriched from CRISPR-Cas9 KO library-transduced HEK293T cells by negative selection using Omicron RBD-Fc (Fig. 2B). Several single guide RNA (sgRNA) sequences were obtained from cells that had lost the ability to bind to Omicron RBD-Fc. Among the genes expressed in the HEK293T cells, we observed the presence of genes involved in the synthesis of glycosaminoglycans (GAGs), such as *SLC35B2* and *B3GAT3* (Fig. 2C). GAGs are highly sulfated polysaccharides covalently attached to the core protein of proteoglycans. SLC35B2 is a transporter of 3'-phosphoadenosine-5'-phosphosulfate, a substrate for the sulfation of GAGs, from the cytosol to the Golgi lumen (26) (Fig. 2D). On the other hand, B3GAT3 is involved in the biosynthesis of the initial common tetrasaccharide structure of a class of GAGs, including HS, chondroitin sulfate (CS), and dermatan sulfate (DS) (27) (Fig. 2D).

In order to analyze the involvement of SLC35B2 or B3GAT3 in the binding of BA.1 spike protein to the cell surface, we generated *SLC35B2* or *B3GAT3* KO HEK293T cells. We found that the binding of BA.1 RBD-Fc to mock HEK293T cells was almost completely lost by deletion of the *SLC35B2* or *B3GAT3* gene, along with cell surface GAGs, including HS and CS (Fig. 2E). Next, the involvement of GAGs in the binding of BA.1 RBD-Fc to the cell surface was analyzed. Cell surface HS, CS, or DS was removed from *ACE2* KO HEK293T cells using heparinase or chondroitinase. BA.1 RBD, but not WT RBD, effectively bound to *ACE2* KO cells. Heparinase treatment, which degrades cell surface HS, significantly reduced the binding of BA.1 RBD to *ACE2* KO HEK293T cells (Fig. 2F). On the other hand, heparinase treatment showed only a marginal effect on the binding of PILRα, a receptor for sialic acids on the *O*-glycan structure (28–30). BA.1 RBD binding was slightly affected by chondroitinase treatment, which degrades cell surface CS and DS. In addition, BA.1 RBD binding was competitively blocked by heparin, an HS analog, in a dose-dependent manner (Fig. 2G), suggesting that BA.1 RBD binds to cell surface HS. Indeed, HS is detected in human nasal tissue (Fig. 2H), where the Omicron variant preferentially infects. We also confirmed that the BA.1 RBD, but not the WT RBD, bound to most of the ACE2-negative cell lines expressing HS (Fig. 2I). These data demonstrate that the BA.1 RBD has acquired the enhanced HS binding that allows the BA.1 spike protein to bind to the cell surface of most cells in an ACE2-independent manner. Although SLC35B2 also affects the sulfation of HS, we used *B3GAT3* KO cells for subsequent analyses because B3GAT3 is more specifically involved in the HS synthesis than SLC35B2.

### The mutations in the RBD acquired by the Omicron BA.1 variant increase the binding of HS to the spike protein

**Fig 3:**
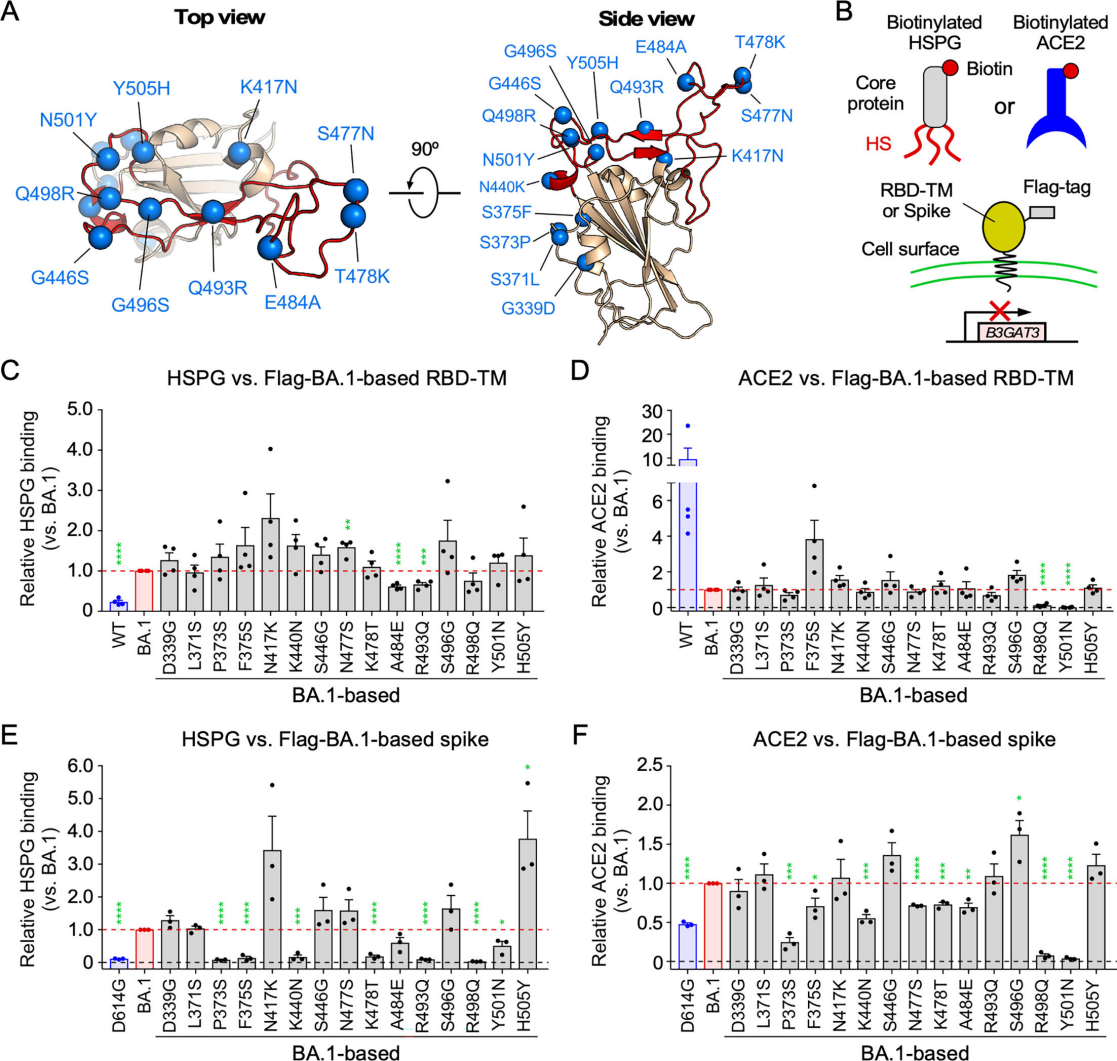
Enhanced binding of RBD to cell surface HS by the mutations acquired by the Omicron variant. (**A**) Structure of BA.1 RBD (PDB: 7WBP) with mutation sites compared to WT (blue spheres) and ACE2-binding sites (receptor-binding motif; red). (**B**) Schematic of the binding assay using biotinylated HSPG or ACE2 with *B3GAT3* KO HEK293T cells expressing Flag-tagged RBD fused to a transmembrane domain (Flag-RBD-TM) or Flag-tagged whole spike protein (Flag-spike). (**C and D**) The binding of biotinylated HSPG (**C**) or ACE2 (**D**) to Flag-RBD-TM transfectants of BA.1-based revertants is shown. Each revertant contains a single mutation reverted to the WT sequence. The expression levels of Flag-RBD-TM were adjusted by anti-Flag Ab staining. Data were normalized to the binding of BA.1. The dashed horizontal red lines indicate the value of parental BA.1. (**E and F**) The binding of biotinylated HSPG (**E**) or ACE2 (**F**) to Flag-spike transfectants of BA.1-based revertants is shown. The expression levels of Flag-spike were adjusted by anti-Flag Ab staining. Data are mean ± SEM of three to four biological replicates. Each dot represents one independent experiment. Statistical analysis was performed using unpaired two-tailed Student’s *t*-tests between parental BA.1 and each revertant; *P < 0.05, **P < 0.01, ***P < 0.001, and ****P < 0.0001.

**Fig 4:**
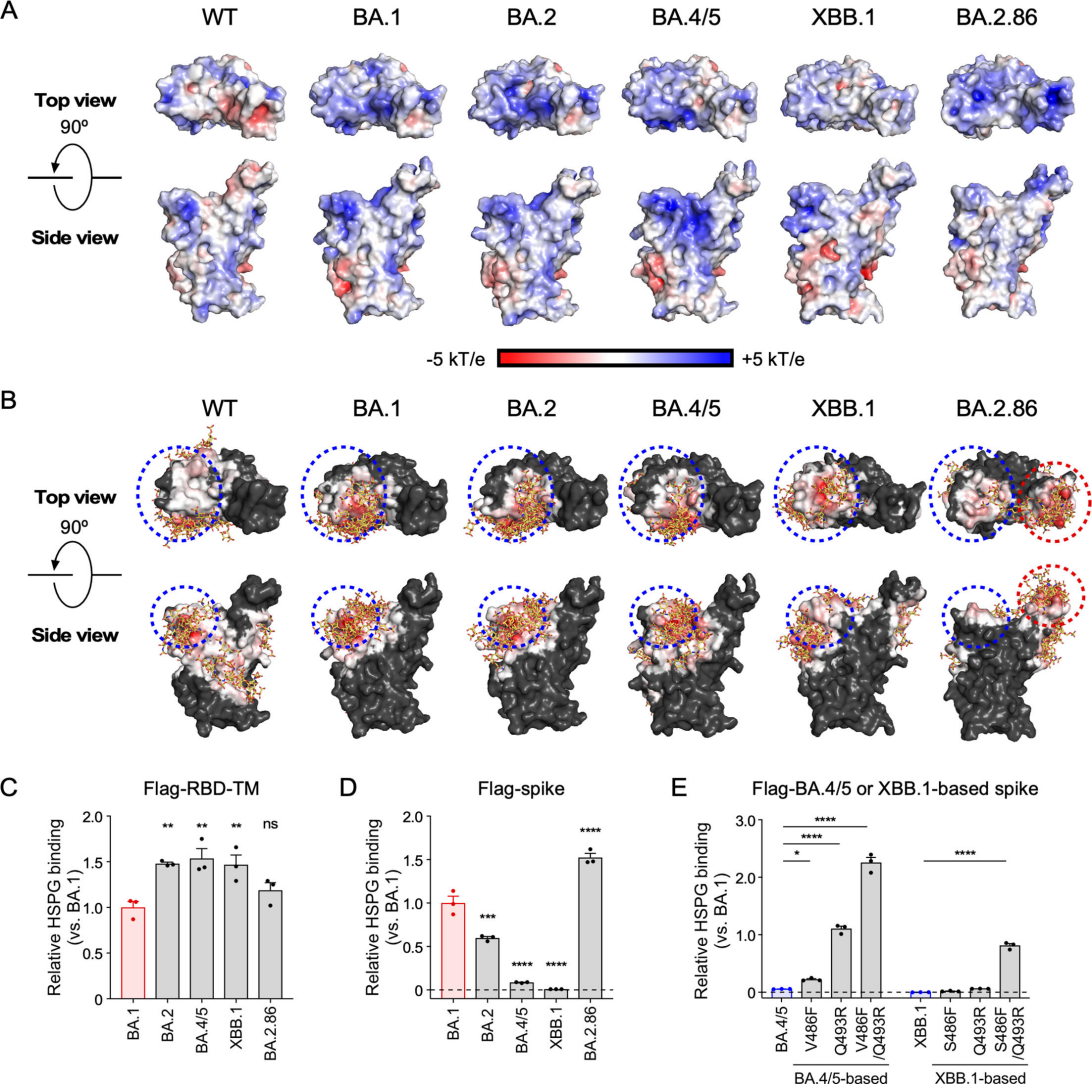
The binding manner of spike protein to HS among the Omicron subvariants. (**A**) Electrostatic potential maps for WT (PDB: 6M0J), BA.1 (7WBP), BA.2 (7XB0), BA.4/5 (7XWA), XBB.1 (8IOV), and BA.2.86 (8QSQ) RBDs are shown. Blue and red colors indicate electropositive and electronegative surfaces, respectively. (**B**) Contact maps showing interactions with 10 predicted heparin poses (yellow sticks) docked to the RBDs of WT, BA.1, BA.2, BA.4/5, XBB.1, and BA.2.86. Red indicates regions with frequent contacts, white indicates fewer contacts, and black indicates no contacts. Blue and red dotted circles indicate putative heparin-binding sites I and II, respectively. (**C and D**) The binding of biotinylated HSPG to *B3GAT3* KO HEK293T cells expressing Flag-RBD-TM (**C**) or Flag-spike (**D**) from BA.1, BA.2, BA.4/5, XBB.1, or BA.2.86. (**E**) The binding of biotinylated HSPG to *B3GAT3* KO HEK293T cells expressing Flag-spike of BA.4/5- or XBB.1-based revertants. Each revertant contains a single or double mutation reverted to the BA.1 sequence. The expression levels of Flag-RBD-TM or Flag-spike were adjusted by anti-Flag Ab staining. Data were normalized to BA.1 binding. Data are mean ± SEM of three technical replicates. Statistical analysis was performed using two-way ANOVA with Dunnett’s multiple comparison test compared to BA.1 in panels **C** and **D** and two-way ANOVA with Dunnett’s multiple comparison test compared to parental BA.4/5 or XBB.1 in panel **E**; *P < 0.05, **P < 0.01, and ****P < 0.0001; ns, not significant. Data are representative of three independent experiments.

The BA.1 subvariant contains 15 mutations in the RBD compared to the WT RBD (Fig. 3A). To identify which mutation in the RBD is involved in HS binding, the BA.1 RBD mutations were reverted to WT RBD sequences, and the binding of the revertants to soluble heparan sulfate proteoglycan (HSPG) or soluble ACE2 was analyzed (Fig. 3B). To avoid the effect of cell surface HS on HSPG or ACE2 binding, we used *B3GAT3* KO HEK293T cells transfected with either Flag-tagged RBD fused to the transmembrane and cytoplasmic domains of PILRα (29, 31) (Flag-RBD-TM) or Flag-tagged whole spike protein (Flag-spike). When the binding of cell surface RBD to soluble HSPG was analyzed, it was observed that the BA.1 RBD exhibited enhanced HS binding compared to the WT RBD (Fig. 3C). The enhanced binding of the BA.1 RBD to HSPG was partially decreased by some reverse mutations, such as A484E and R493Q, suggesting that the E484A and Q493R mutations increase the HS binding of the BA.1 RBD (Fig. 3C). On the other hand, the R498Q and Y501N revertants decreased ACE2 binding, and the F375S and S496G revertants increased ACE2 binding compared to the parental BA.1 RBD (Fig. 3D), which is consistent with previous reports (14–16). Moreover, the BA.1 spike demonstrated enhanced HS binding compared to the D614G spike (Fig. 3E). Interestingly, the HSPG binding of the BA.1 spike was almost completely abolished by the P373S, F375S, K440N, K478T, R493Q, and R498Q revertants (Fig. 3E). Conversely, the binding of the BA.1 spike to HSPG was enhanced by the H505Y revertant compared to the parental BA.1 spike. Similar to the results observed in Fig. 3D, the binding of BA.1 spike to ACE2 is decreased by the R498Q and Y501N revertants and increased by the S496G revertant compared to the parental BA.1 spike (Fig. 3F). Among the mutations that affect the binding of HSPG to the BA.1 RBD or spike protein, glutamic acid (E), arginine (R), and lysine (K) are charged amino acids, and the N440K, T478K, E484A, Q493R, and Q498R mutations increase the positive electrostatic surface potential on the RBD of the BA.1 spike (Fig. 4A). As HS is a negatively charged polysaccharide (Fig. 2D), it is likely that the positive charge acquired by the BA.1 RBD is involved in the enhanced binding to HS.

### The binding mode of spike protein to HS diverges among the Omicron subvariants

When we investigated the electrostatic surface potential on the RBD of the Omicron subvariants, such as BA.1, BA.2, BA.4/5 (BA.4 and BA.5), XBB.1, and BA.2.86, the positively charged area on the Omicron RBD was increased compared to that on the WT RBD (Fig. 4A), suggesting that Omicron subvariants other than BA.1 also bind to HS. To predict the HS binding site on the RBD, we analyzed the heparin-binding sites on the RBD using the docking server ClusPro (32–36). The heparin-binding sites of the Omicron variants were extended to the ACE2-binding sites (receptor-binding motif), compared to those of the WT SARS-CoV-2. Mutations to positively charged amino acids in BA.1, such as Q493R and Q498R, are likely to be involved in the extension of the heparin-binding sites observed in the BA.1 RBD (Fig. 4B), which is consistent with the results observed in Fig. 3C and E. In addition, the heparin-binding sites of Omicron subvariants, such as BA.1, BA.2, BA.4/5, and XBB.1, are located at the upper left of the RBD (putative heparin-binding region I; Fig. 4B), whereas the heparin-binding sites of the recent Omicron subvariant BA.2.86 are mainly located at the upper right of the RBD (putative heparin-binding region II; Fig. 4B), suggesting that HS binding sites have shifted during the evolution of the Omicron lineage.

We analyzed the binding of HS to the cell surface RBD or whole spike protein of the Omicron subvariants BA.1, BA.2, BA.4/5, XBB.1, and BA.2.86. HSPG bound efficiently to the BA.2, BA.4/5, XBB.1, and BA.2.86 RBDs, similar to the BA.1 RBD (Fig. 4C). On the other hand, the specificity of HSPG binding to the whole spike protein was different from that to the RBD. HSPG bound to the BA.1, BA.2, and BA.2.86 spike proteins on the cell surface, but not to the BA.4/5 and XBB.1 spike proteins, although HSPG efficiently bound to BA.4/5 and XBB.1 RBDs (Fig. 4D). The spike protein is a trimer, and each RBD exhibits the open “up” or closed “down” conformation (37, 38). It has been reported that one of the three RBDs of the BA.1 (39, 40), BA.2 (40), and BA.2.86 (41) spike proteins is in the up conformation. In contrast, the three RBDs of the BA.4/5 (40) and XBB.1 (22) spike proteins are mostly in the down conformation in the steady state. Some mutations, such as S373P, S375F, Q493R, and Q498R, are implicated in stabilizing the one-up RBD conformation of BA.1 (39, 42, 43), and their revertants abolished the binding of HSPG to the BA.1 spike protein (Fig. 3E). The mutations acquired by the BA.4/5 and XBB.1 variants appear to be involved in the down conformation of the RBD. Both variants harbor mutations at the amino acid residues that are involved in stabilizing the up conformation of the BA.1 RBD. In particular, the F486V (in BA.4/5) or F486S (in XBB.1) mutation, along with the R493Q mutation (present in both BA.4/5 and XBB.1), is thought to contribute to the predominantly closed conformation of the BA.4/5 and XBB.1 spike proteins (39, 42, 43). We found that reverting these mutations in the BA.4/5 and XBB.1 variants to the corresponding BA.1 residues enhanced the binding of soluble HSPG to the BA.4/5 and XBB.1 spike proteins (Fig. 4E). Consequently, the HS binding sites in the BA.4/5 and XBB.1 spike proteins appear to be closed in the steady state. The binding of HS to the spike protein seems to be regulated not only by the affinity to RBD but also by the conformation of RBD.

### Anti-RBD neutralizing Abs from Omicron BA.1-infected patients block the interaction between Omicron spike and HS

**Fig 5:**
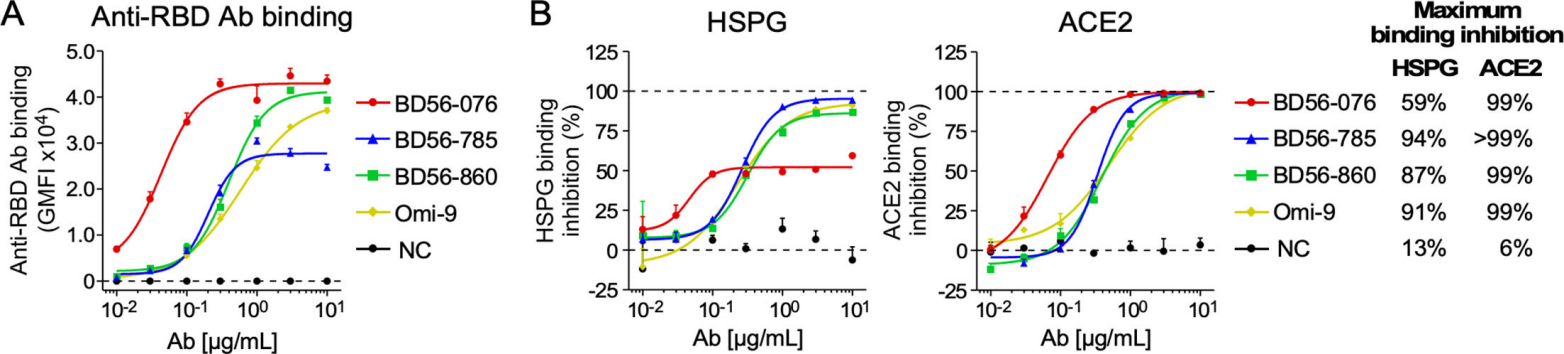
Inhibition of HS binding to Omicron BA.1 spike by anti-RBD neutralizing Abs from BA.1-infected patients. (**A**) Binding of anti-RBD Abs from BA.1-infected patients to BA.1 spike. *B3GAT3* KO HEK293T cells lacking HS were transfected with BA.1 spike and were used for Ab binding. (**B**) Inhibition of HSPG or ACE2 binding to BA.1 spike by anti-RBD Abs. HSPG or ACE2 binding to *B3GAT3* KO HEK293T cells transfected with BA.1 spike was analyzed in the presence or absence of anti-RBD Abs. The maximum binding inhibition of Abs for HSPG or ACE2 binding to BA.1 spike is shown. Data are mean ± SEM of three technical replicates. Data are representative of two independent experiments.

Antibodies to RBD play an important role in protecting against SARS-CoV-2 infection by blocking the interaction between ACE2 and RBD. We analyzed anti-RBD Abs (BD56-076, BD56-785, BD56-860, and Omi-9) from BA.1-infected patients (40, 44) to test whether anti-RBD Abs are involved in blocking the interaction between HS and the BA.1 spike protein. To avoid the effect of cell surface HS on Ab binding, we transfected BA.1 spike to *B3GAT3* KO HEK293T cells that lack cell surface HS. All anti-RBD Abs efficiently bound to BA.1 spike transfectants (Fig. 5A). Some anti-RBD Abs not only inhibited the binding of ACE2 to BA.1 spike protein but also inhibited that of HSPG (Fig. 5B). Interestingly, BD56-076 Ab completely blocked the binding to ACE2 but only partially blocked the binding to HSPG. This suggests that the HS binding sites on the BA.1 RBD are different from those of ACE2, although the HS binding sites are adjacent to the ACE2 binding sites.

### Cell surface HS is required for infection of low-level ACE2-expressing cells by the Omicron variants

**Fig 6:**
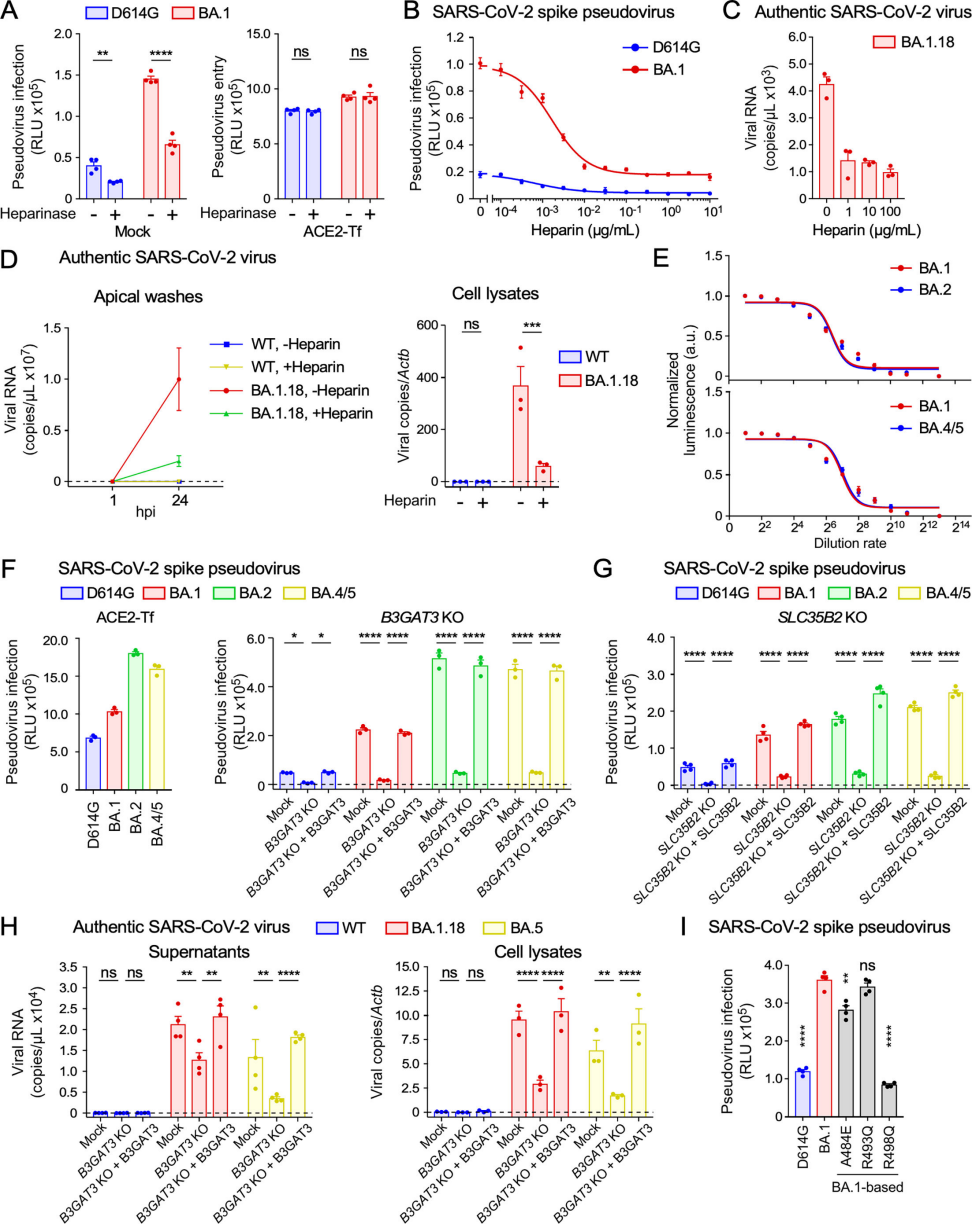
HS-dependent infection of the Omicron variants to low-level ACE2-expressing cells. (**A**) Infection of D614G or BA.1 pseudovirus to mock or ACE2-Tf HEK293T cells pretreated with (+) or without (–) heparinase. (**B**) Infection of D614G or BA.1 pseudovirus to HEK293T cells at different heparin concentrations. (**C**) Infection of authentic SARS-CoV-2 BA.1.18 variant to HEK293T cells at different heparin concentrations. Viral RNA in the supernatants at 24 hours post-inoculation (hpi) is shown. (**D**) Infection of authentic SARS-CoV-2 WT or BA.1.18 variant to primary human nasal epithelial cells in the presence (+) or absence (–) of 100 µg/mL heparin. Viral RNA in apical washes at 1 and 24 hpi or in cell lysates at 24 hpi is shown. Lysate RNA was normalized to *Actb*. (**E**) Titration of BA.2 or BA.4/5 pseudovirus using VSV-G-Tf HEK293T cells. (**F**) Infection of D614G, BA.1, BA.2, or BA.4/5 pseudovirus to ACE2-Tf HEK293T cells or to mock, *B3GAT3* KO, or B3GAT3-Tf *B3GAT3* KO HEK293T cells. (**G**) Infection of D614G, BA.1, BA.2, or BA.4/5 pseudovirus to mock, *SLC35B2* KO, or SLC35B2-Tf *SLC35B2* KO HEK293T cells. (**H**) Infection of authentic SARS-CoV-2 WT, BA.1.18 or BA.5 variant to mock, *B3GAT3* KO, or B3GAT3-Tf *B3GAT3* KO HEK293T cells. Viral RNA in supernatants or cell lysates at 24 hpi is shown. Lysate RNA was normalized to *Actb*. (**I**) Infection of D614G, BA.1, or BA.1-based revertant (A484E, R493Q, and R498Q) pseudovirus to HEK293T cells. Data are mean ± SEM of three to four technical replicates. Statistical analysis was performed using two-way ANOVA with Sidak’s multiple comparison tests in panels **A** and **D**, two-way ANOVA with Dunnett’s multiple comparison test compared to *B3GAT3* KO HEK293T cells in panels **F** and **H**, two-way ANOVA with Dunnett’s multiple comparison test compared to *SLC35B2* KO HEK293T cells in panel **G**, and unpaired two-tailed Student’s *t*-tests between parental BA.1 and each revertant in panel **I**; *P < 0.05, **P < 0.01, ***P < 0.001, and ****P < 0.0001; ns, not significant. Data are representative of two to three independent experiments.

We investigated the effect of cell surface HS on SARS-CoV-2 infection. The infectivity of the BA.1 pseudovirus to mock HEK293T cells expressing low levels of ACE2 was significantly reduced by heparinase treatment (Fig. 6A). Although the infectivity of the D614G pseudovirus was also slightly reduced by heparinase treatment, the overall infectivity of the D614G pseudovirus to mock HEK293T cells was quite low. On the other hand, the infectivity of both BA.1 and D614G pseudoviruses to ACE2-transfected HEK293T cells was not affected by heparinase treatment. This suggests that the binding of the spike protein to HS is crucial for the infection of the BA.1 variant to low-level ACE2-expressing cells. Furthermore, we confirmed that BA.1 pseudovirus infection of HEK293T cells was inhibited by heparin in a dose-dependent manner (Fig. 6B). Consistent with the results obtained using the pseudoviruses, infection of HEK293T cells by the authentic SARS-CoV-2 BA.1.18 variant was also inhibited by heparin (Fig. 6C). Notably, the authentic BA.1.18 variant exhibited significantly higher infectivity in primary human nasal epithelial cells compared to the WT virus (Fig. 6D). Furthermore, infection of primary human nasal epithelial cells by the authentic BA.1.18 variant was effectively inhibited by heparin. These data suggest that the BA.1.18 variant efficiently utilizes HS for infection under physiological conditions.

To analyze whether Omicron variants other than BA.1 also utilize HS for infection, we prepared pseudoviruses of Omicron BA.2 and BA.4/5 with titers almost the same as the BA.1 pseudovirus (Fig. 6E). The BA.2 and BA.4/5 pseudoviruses showed higher infectivity to mock and ACE2-Tf HEK293T cells compared to D614G and BA.1 pseudoviruses (Fig. 6F). The enhanced infectivity of the Omicron (BA.1, BA.2, and BA.4/5) pseudoviruses to mock HEK293T cells was reduced by deletion of the *B3GAT3* gene, which abrogates cell surface HS. Transfection of the *B3GAT3* gene into *B3GAT3* KO cells restored the infectivity of the pseudoviruses (Fig. 6F). Similarly, knockout of the *SLC35B2* gene in mock HEK293T cells attenuated the enhanced infectivity of Omicron (BA.1, BA.2, and BA.4/5) pseudoviruses (Fig. 6G). As with the Omicron pseudovirus, the high infectivity of authentic SARS-CoV-2 Omicron (BA.1.18 and BA.5) variants to mock HEK293T cells was decreased by the knockout of *B3GAT3* and restored by introducing the *B3GAT3* gene (Fig. 6H), indicating that cell surface HS is required to infect low-level ACE2-expressing cells. Mutations E484A, Q493R, and Q498R are implicated in the high HS binding affinity of BA.1 (Fig. 3C). To investigate the impact of these mutations on the elevated infectivity of BA.1 to cells expressing low levels of ACE2, we generated BA.1 pseudoviruses in which these residues were reverted to those of the WT virus. Infection of HEK293T cells with the BA.1 pseudovirus was significantly reduced when A484 and R498 of BA.1 were reverted to WT E484 and Q498, respectively (Fig. 6I). These findings suggest that the Omicron variant has acquired infectivity to low-level ACE2-expressing cells through mutations that enhance HS binding.

### TMPRSS2 reduces the level of cell surface HS

TMPRSS2 cleaves the SARS-CoV-2 spike protein, which plays an important role in the membrane fusion process during SARS-CoV-2 infection (17). On the other hand, it has been reported that the Omicron variant shows low infectivity to the TMPRSS2-expressing cells compared to the earlier variants (18–20), although the molecular mechanism underlying the low infectivity to TMPRSS2-expressing cells remains unclear. TMPRSS2 cleaves the C-terminus of basic amino acids, arginine or lysine residues, similar to trypsin (45, 46). Interestingly, cell surface proteoglycans have been reported to be cleaved by trypsin (47), suggesting that the amount of cell surface HS may be reduced by TMPRSS2.

**Fig 7:**
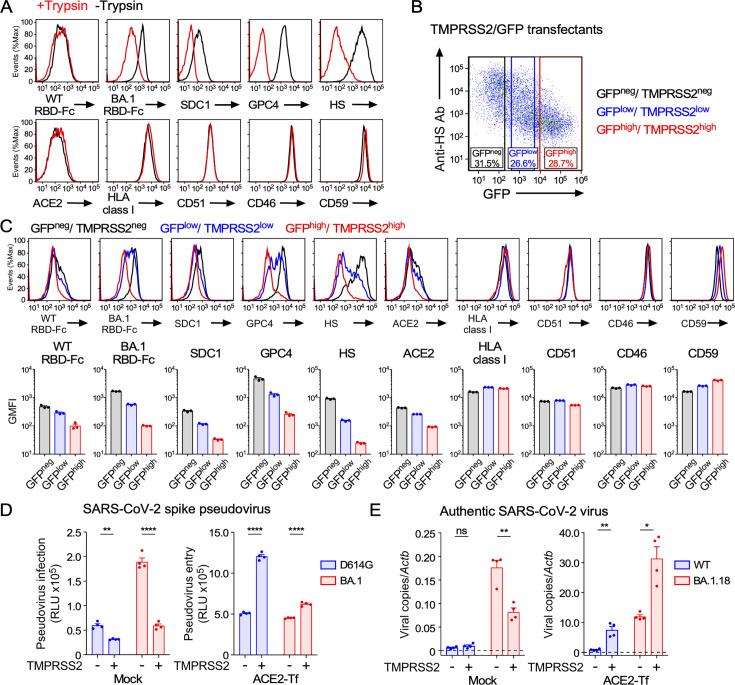
TMPRSS2 cleaves cell surface HS proteoglycans. (**A**) Binding of WT or BA.1 RBD-Fc, anti-syndecan-1 (SDC1) Ab, anti-glypican-4 (GPC4) Ab, anti-HS Ab, anti-ACE2 Ab, anti-HLA class I Ab, anti-integrin αV (CD51) Ab, anti-CD46 Ab, or anti-CD59 Ab to HEK293T cells pretreated with (red line) or without (black line) trypsin. (**B**) Representative gating strategy for TMPRSS2-negative (GFP^neg^, black), low-expressing (GFP^low^, blue), or high-expressing (GFP^high^, red) cells in TMPRSS2/GFP-Tf HEK293T cells. (**C**) Top: representative histograms showing the binding of WT or BA.1 RBD-Fc, anti-SDC1 Ab, anti-GPC4 Ab, anti-HS Ab, anti-ACE2 Ab, anti-HLA class I Ab, anti-CD51 Ab, anti-CD46 Ab, or anti-CD59 Ab to TMPRSS2-negative (black line, GFP^neg^), low-expressing (blue line, GFP^low^) or high-expressing (red line, GFP^high^) cells. Bottom: quantification of binding is shown as GMFI. (**D**) Infection of D614G or BA.1 pseudovirus to mock or ACE2-Tf HEK293T cells with (+) or without (–) TMPRSS2 expression. (**E**) Infection of authentic SARS-CoV-2 WT or BA.1.18 variant to mock or ACE2-Tf HEK293T cells with (+) or without (–) TMPRSS2 expression. Viral RNA in cell lysates at 24 hours post-inoculation is shown. Lysate RNA was normalized to *Actb*. Data are mean ± SEM of three to four technical replicates. Statistical analysis was performed using two-way ANOVA with Sidak’s multiple comparison tests in panel **D** and unpaired two-tailed Welch’s *t*-tests between WT and BA.1.18 in panel **E**; *P < 0.05, **P < 0.01, and ****P < 0.0001; ns, not significant. Data are representative of two to three independent experiments.

When HEK293T cells were treated with trypsin, the expression levels of cell surface HSPGs, such as syndecan-1 (SDC1) and glypican-4 (GPC4), were dramatically decreased, whereas the expression levels of most cell surface molecules, such as ACE2, HLA class I, integrin αV (CD51), CD46, and CD59, were not affected at all (Fig. 7A), indicating that specific cell surface proteins were cleaved by trypsin. We confirmed that most of the cell surface HS was removed by trypsin treatment. Accordingly, the binding of BA.1 RBD-Fc to the cell surface was also reduced by trypsin treatment. Given the similarity in specificity between trypsin and TMPRSS2 (45, 46), it is possible that TMPRSS2 also cleaves cell surface HSPGs. We analyzed TMPRSS2 high- and low-expressing populations in HEK293T cells co-transfected with TMPRSS2 and green fluorescent protein (GFP) by gating based on GFP expression (Fig. 7B). When TMPRSS2 was transfected into HEK293T cells, BA.1 RBD-Fc binding to the cell surface was dramatically reduced, along with a decrease in cell surface SDC1, GPC4, and HS, even on the cells expressing low levels of TMPRSS2 (Fig. 7C). Although cell surface ACE2 was decreased on cells expressing high levels of TMPRSS2, as previously reported (48), the decrease in ACE2 expression was small in cells expressing low levels of TMPRSS2. In contrast, the expression levels of most cell surface molecules, such as HLA class I, CD51, CD46, and CD59, were not affected at all, even though TMPRSS2 was expressed at high levels. These data indicate that TMPRSS2 is involved in the regulation of cell surface HS levels.

Finally, we analyzed the infectivity of the Omicron variant to TMPRSS2 transfectants. TMPRSS2 expression significantly enhanced the infectivity of the D614G pseudovirus to ACE2-transfected HEK293T cells. On the other hand, the infectivity of BA.1 pseudovirus to mock HEK293T cells expressing low levels of ACE2 was significantly reduced by TMPRSS2 expression (Fig. 7D), suggesting that TMPRSS2 expression reduces HS-dependent viral entry. Similar to the pseudovirus analyses, TMPRSS2 expression significantly reduced the infectivity of the authentic SARS-CoV-2 BA.1.18 variant in mock HEK293T cells expressing low levels of ACE2 (Fig. 7E). Given that HS is required for Omicron infection of ACE2 low-expressing cells, these results suggest that TMPRSS2-mediated cleavage of HSPGs may contribute to the reduced infectivity of the Omicron variant to TMPRSS2-expressing cells.

## DISCUSSION

The Omicron variant has acquired a number of mutations in the RBD that contribute to escape from neutralizing Abs (11–13). To investigate the impact of these mutations, we conducted a library screening for molecules that bind to the Omicron RBD and identified *SLC35B2* and *B3GAT3* as candidate genes. *SLC35B2* encodes a transporter for the sulfate donor required for sulfation (26), whereas *B3GAT3* encodes a glycosyltransferase involved in an early step of HS biosynthesis (27). We found that the Omicron RBD exhibits enhanced binding to HS as demonstrated by inhibition experiments using heparin and heparinase treatment. Therefore, although both SLC35B2 and B3GAT3 are involved in HS synthesis, we chose to use *B3GAT3* KO cells in our analysis to more specifically evaluate the role of HS in viral infection. In addition, as HS is a highly negatively charged molecule, mutations to positively charged amino acid residues resulted in increased binding of the Omicron spike protein to cell surface HS. Although the WT spike protein has been reported to bind to HS (8), the WT RBD showed little binding to cell surface HS. This indicates that the Omicron spike protein has acquired a much higher binding capacity to HS than the WT spike protein, suggesting that the Omicron variant increases virus binding to the cell surface. Indeed, we observed that the Omicron variant infects low-level ACE2-expressing cells and primary human nasal epithelial cells in an HS-dependent manner, whereas WT SARS-CoV-2 barely infects these cells. We also confirmed that the infectivity of the Omicron variant to cells expressing low levels of ACE2 was reduced by reverting key mutations involved in HS binding to the WT sequences. Our results demonstrate that enhanced HS binding promotes Omicron entry; however, in tissues where ACE2-expressing cells are scarce, increased HS binding may inhibit infection by sequestering viral particles away from these target cells. Previous studies have reported that the proportion of ACE2-expressing cells is lower in the lung than in the nasal tissue (49, 50). Thus, while enhanced HS binding of the Omicron variant may facilitate viral entry into cells in the nasal tissue, it may reduce viral infection of cells in the lung. Indeed, it has been reported that the Omicron variant preferentially infects the upper respiratory tract, compared to the earlier variants (51, 52). As viral tropism is considered a key factor in disease pathogenesis, enhanced HS binding may influence the pathogenic characteristics of the Omicron variant.

The binding of HSPG to whole spike proteins of BA.4/5 and XBB.1 was low compared to that to BA.1, BA.2, and BA.2.86 spike proteins, although HSPG bound efficiently to RBDs of BA.4/5 and XBB.1. It has been reported that most of the RBDs of BA.4/5 (40) and XBB.1 (22) spike proteins are present in a down conformation, whereas the RBDs of BA.1 (39, 40), BA.2 (40), and BA.2.86 (41) spike proteins tend to be in an up conformation in the steady state. Notably, HS binding of the BA.4/5 and XBB.1 spike proteins was enhanced by introducing mutations that are thought to stabilize the up (open) conformation of the BA.1 RBD. These findings suggest that the reduced HS binding observed in the BA.4/5 and XBB.1 spike proteins is attributable to their predominantly closed conformation. Thus, the open/closed conformational state of the spike protein appears to play a critical role in modulating HS binding. On the other hand, BA.4/5 exhibits high HS-dependent infectivity, suggesting that the up conformation of the BA.4/5 and XBB.1 spike protein RBDs is likely to be induced during infection. The strong affinity of viral envelope proteins to cell surface molecules not only enhances viral infection but also blocks virus release from the cell surface. In the case of influenza viruses, viral neuraminidase plays an important role in virus release from cells by promoting virus detachment from the cell surface (53). Therefore, the down conformation of the RBD in the steady state observed for the BA.4/5 and XBB.1 spike proteins might be involved in the high virus production from cells.

HS serves as an attachment factor for various human viruses, such as herpes simplex viruses (54), dengue virus (55, 56), hepatitis B virus (57–59), and RS virus (60, 61). Some viruses have been reported to acquire HS binding through repeated passages (62, 63), suggesting that acquisition of HS binding is one of the processes of adaptation to humans. We demonstrated that the RBD of Omicron subvariants conserves high HS binding ability. In addition, docking simulations of heparin to the RBD revealed that the putative heparin-binding sites on the RBD differ among Omicron subvariants, particularly in the later Omicron subvariant BA.2.86. These findings suggest that while HS plays a role in Omicron infection, the role of HS may not be the same among SARS-CoV-2 variants.

We showed that almost all cell surface HSPGs are cleaved by TMPRSS2, whereas most cell surface molecules are resistant to TMPRSS2. Syndecan and glypican are the major cell surface HSPGs (64), and both HSPGs have large unstructured regions accessible to glycosyltransferases (AlphaFold Protein Structure Database; https://alphafold.ebi.ac.uk/), which are also thought to be accessible to proteases. Since TMPRSS2 is highly expressed in alveolar epithelial cells of the lung (50, 65), infectivity in TMPRSS2-expressing cells is thought to be associated with SARS-CoV-2 pathogenicity. Indeed, wild-type SARS-CoV-2 has been reported to preferentially infect TMPRSS2-expressing cells, whereas the dependency on TMPRSS2 is reduced in the Omicron variant (18–20). This relatively low infectivity of Omicron in TMPRSS2-expressing cells may, in part, be attributable to reduced viral attachment resulting from TMPRSS2-mediated cleavage of HSPGs. However, as most of the cell lines exhibit low TMPRSS2 activity, we were unable to elucidate the function of TMPRSS2 under more physiological conditions. Further studies on the effect of TMPRSS2 on cell surface HSPGs will help to elucidate the detailed mechanism of SARS-CoV-2 infection.

## MATERIALS AND METHODS

### Cell lines

HEK293T cells (RIKEN Cell Bank), ACE2-stably expressing HEK293T cells (previously prepared [66]), NCI-H1299 cells (ATCC), HT29 cells (ATCC), HeLa cells (ATCC), and VeroE6-TMPRSS2 cells (JCRB Cell Bank) were cultured in Dulbecco’s modified Eagle’s medium (DMEM; Nacalai Tesque) supplemented with 10% fetal bovine serum (FBS) and 1% penicillin-streptomycin (PS; Nacalai Tesque). A549 cells (ATCC) were cultured in Roswell Park Memorial Institute medium (Nacalai Tesque) supplemented with 10% FBS and 1% PS. HAP1 cells (a gift from Dr. Maeda [67]) were cultured in Eagle’s minimum essential medium (Fujifilm) supplemented with 10% FBS and 1% PS. Expi293 cells (Thermo Scientific) were cultured in Expi293 medium. All cell lines were cultured at 37°C under 5% CO_2_ except for Expi293 cells, which were cultured at 37°C under 8% CO_2_.

### Viruses

The SARS-CoV-2 wild-type strain (WT; B lineage: SARS-CoV-2/Hu/DP/Kng/19-020, GenBank accession number: LC528232) was obtained from Kanagawa Prefectural Institute of Public Health (Kanagawa, Japan). The Omicron BA.1 subvariant (BA.1.18 lineage: hCoV-19/Japan/TY38-873/2021) and BA.5 subvariant (GISAID accession number: EPI_ISL_13241867) were obtained from the National Institute of Infectious Diseases (Tokyo, Japan). To prepare the working virus, 50 µL of the virus was inoculated into 2 mL of ACE2-stably expressing HEK293T cells (10,000,000 cells in a 10 cm dish). At 20 min after infection, 6–8 mL DMEM with 10% FBS and 1% PS was added. At 2 days post-infection, the culture medium was collected and centrifuged at 300 × *g* for 10 min at 4°C. Aliquots of the virus were stored at −80°C until use. To determine virus titers, serially diluted viruses were inoculated into VeroE6-TMPRSS2 cells and incubated at 37°C for 3–4 days. The 50% tissue culture infectious dose (TCID_50_) was determined by observing the cytopathic effect under a microscope. The TCID_50_/mL was calculated according to the Reed-Muench method (68). The SARS-CoV-2 infection assay was performed in a biosafety level 3 laboratory.

### Plasmid construction

Plasmids for the SARS-CoV-2 D614G spike (containing only the D614G mutation) gene lacking 19 C-terminal amino acids (amino acids 1–1,254) were cloned into a pME18S expression vector as previously described (66). For the production of the pseudovirus, the truncated spike gene (amino acids 1–1,254) was cloned into a pcDNA3.4 expression vector. For HSPG and ACE2 binding assays, the spike gene (amino acids 14–1,254) was cloned into a pME18S expression vector containing N-terminal BM40 signal and Flag-tag sequences. Furthermore, the RBD (amino acids 335–587) of the spike gene was cloned into a pME18S expression vector containing N-terminal SLAM signal and Flag-tag sequences and the C-terminal transmembrane and cytoplasmic domains of PILRα as previously reported (29, 31). In addition, a series of mutated Flag-tagged BA.1 spike or Flag-tagged RBD fused to the transmembrane and cytoplasmic domains of mouse PILRα were prepared using a QuickChange Lighting Multi Site-directed Mutagenesis Kit. The primers for mutagenesis were designed on the Agilent website (https://www.agilent.com/store/primerDesignProgram.jsp). For RBD fused with human IgG1 (RBD-Fc) production, the RBD (amino acids 335–587) of the spike gene was cloned into a pcDNA3.4 expression vector encoding the N-terminal SLAM signal sequence and C-terminal human IgG1 Fc sequence. Omicron BA.1, BA.2, BA.4/5, and BA.2.86 lineages were prepared from the SARS-CoV-2 spike gene using a QuickChange Lightning Multi Site-directed Mutagenesis Kit (Agilent, 210513). A series of mutated Flag-tagged BA.4/5 or XBB.1 spike was prepared using a QuickChange Lighting Multi Site-directed Mutagenesis Kit. The cDNA encoding human TMPRSS2 (BC051839) was cloned into a pME18S expression vector. For the production of anti-RBD monoclonal Abs, genes for variable regions of the Abs from BA.1-infected patients were synthesized according to the published sequence (40, 44) (Integrated DNA Technologies). The cDNA sequences of the variable regions of the heavy chain linked to that of the light chain with the RGSTSGSGKPGSGEGS linker sequence were cloned into a pcDNA3.4 expression vector containing the human IgG1 Fc sequence. The DNA sequences of these constructs were confirmed by sequencing (ABI3130xl).

### Transfection

Transient transfection of HEK293T cells was performed using Lipofectamine LTX Reagent (Thermo Scientific, 1533810) or PEI Max (Polysciences, 26406). Stable transfectants were generated by retrovirus-mediated transduction using the pMxs-puro retroviral expression vector (69). Briefly, the pMxs-puro retroviral expression vector, pantropic envelope VSV-G, and murine leukemia virus (MLV) gag-pol genes were co-transfected into HEK293T cells. Cell culture supernatants containing the retroviruses were harvested 2 days later, and spin transfection was performed at 1,200 × *g* for 2 hours at 32°C.

### Antibodies and recombinant proteins

Mouse anti-human ACE2 monoclonal antibody (mAb) (AC384, Adipogen), mouse anti-HS mAb (F58-10E4, Amzbio), mouse anti-CS mAb (CS-56, Sigma-Aldrich), rat anti-Flag-tag mAb (L5, BioLegend), mouse anti-human CD138 (syndecan-1) mAb (MI15, BioLegend), mouse anti-human glypican-4 mAb (961609, R&D Systems), mouse anti-human CD46 mAb (J4.48, Beckman), mouse anti-human CD59 mAb (p282[H19], BioLegend), mouse anti-human HLA class I mAb (W6/32, Institute of Development, Aging and Cancer, University of Tohoku), mouse anti-human CD51 (integrin αV) mAb (L230, Enzo), Alexa Fluor 555 goat anti-mouse IgM (Heavy chain) Ab (Thermo Scientific), allophycocyanin (APC)-conjugated goat anti-human IgG, Fcγ fragment specific Ab, APC-conjugated goat anti-mouse IgG, Fcγ fragment specific Ab, APC-conjugated goat anti-mouse IgM, µ chain specific Ab, APC-conjugated donkey anti-rat IgG (H + L) Ab, and APC-conjugated streptavidin (Jackson) were used.

The plasmids for the RBD-Fc were transfected into Expi293 cells (Thermo Scientific), and the cell culture supernatants were collected according to the manufacturer’s protocols. The concentration of recombinant RBD-Fc protein in the supernatants was measured using Protein A-coupled latex beads (Thermo Scientific, A37304) and APC-conjugated anti-human IgG Fc Ab (Jackson) against RBD-Fc standards of known concentration as previously reported (66). The expression plasmids for the anti-RBD monoclonal Abs were transfected into Expi293 cells, and the cell culture supernatants were collected and purified using Protein A Sepharose (GE Healthcare, 17127903). Biotinylated ACE2 fused with mouse IgG2a Fc (ACE2-Fc) and cell culture supernatants of mouse PILRα fused with human IgG1 Fc (PILRα-Fc) were prepared as previously described (28, 66).

### Flow cytometric analysis of RBD-Fc binding or binding inhibition by heparin

Cell lines were stained with WT or BA.1 RBD-Fc (10 µg/mL), or mouse anti-ACE2 mAb (5.0 µg/mL) for 30 min at 4°C, followed by detection of RBD-Fc- or mAb-bound cells using APC-conjugated anti-human IgG Fc Ab (1.3 µg/mL) or APC-conjugated anti-mouse IgG Fc Ab (1.3 µg/mL). For the heparin inhibition assay, the WT or BA.1 RBD-Fc (final 5.0 µg/mL) was premixed with heparin (Sigma-Aldrich, H3149) at the indicated concentrations, followed by incubation of HEK293T cells with these mixtures for 30 min at 4°C. Thereafter, RBD-Fc bound to the cell surface was detected using APC-conjugated anti-human IgG Fc Ab (1.3 µg/mL). Dead cells were stained with propidium iodide (PI; Sigma-Aldrich, 25535-16-4). The stained cells in the live cells were analyzed using flow cytometers (FACSVerse, BD biosciences, or Attune, Thermo Scientific). Dose-response curves for the inhibition of RBD-Fc binding to HEK293T cells by heparin were calculated using a four-parameter non-linear regression model with GraphPad Prism version 7.0e.

### Flow cytometric analysis of enzymatically treated cells

For the heparinase or chondroitinase treatment, *ACE2* KO HEK293T cells were pretreated with Heparinase I and III (2 U/mL; Sigma-Aldrich, H3917) in Hanks' balanced salt solution (HBSS) containing 4 mM CaCl_2_, 20 mM 4-(2-hydroxyethyl)-1-piperazineethanesulfonic acid (HEPES), and 0.1% bovine serum albumin (BSA) for 1 hour at 30°C or with Chondroitinase ABC (2 U/mL; Sigma-Aldrich, C2905) in HBSS containing 20 mM HEPES and 0.1% BSA for 1 hour at 37°C, followed by incubation with WT or BA.1 RBD-Fc (10 µg/mL), PILRα-Fc, mouse anti-HS mAb (5.0 µg/mL), or mouse anti-CS mAb (1:100 dilution). Thereafter, WT or BA.1 RBD-Fc-, PILR-Fc-, or mAb-bound cells were detected using APC-conjugated anti-human IgG Fc Ab (1.3 µg/mL) or APC-conjugated anti-mouse IgM Ab (2.5 µg/mL). For trypsin treatment, HEK293T cells were pretreated with 2.5 mg/mL trypsin and 1 mM EDTA solution (Nacalai Tesque) for 5 min at 37°C, followed by incubation with WT or BA.1 RBD-Fc (10 µg/mL), mouse anti-syndecan-1 mAb (5.0 µg/mL), mouse anti-glypican-4 mAb (5.0 µg/mL), mouse anti-HS mAb (5.0 µg/mL), mouse anti-ACE2 mAb (5.0 µg/mL), mouse anti-CD46 mAb (2.0 µg/mL), mouse anti-CD59 mAb (5.0 µg/mL), mouse anti-integrin αV mAb (2.0 µg/mL), or mouse anti-HLA class I mAb (3.3 µg/mL) for 30 min at 4°C. Thereafter, RBD-Fc- or mAb-bound cells were detected using APC-conjugated anti-human IgG Fc Ab (1.3 µg/mL), APC-conjugated anti-mouse IgG Fc Ab (1.3 µg/mL), or APC-conjugated anti-mouse IgM Ab (2.5 µg/mL). Dead cells were stained with PI. The stained cells in the live cells were then analyzed using a flow cytometer (FACSVerse, BD Biosciences, or Attune, Thermo Scientific).

### Generation of knockout cell lines

To generate *ACE2* KO HEK293T cells, the target sequence of ACE2 (5′-ACAGTTTAGACTACAATGAG-3′) was cloned into a pX330 CRISPR KO vector (Addgene), and then this plasmid was co-transfected into HEK293T cells with GFP. After GFP-positive cells were sorted, anti-ACE2 mAb- and GFP-negative cells were obtained using a cell sorter (SH800, Sony). To generate *SLC35B2* or *B3GAT3* KO HEK293T cells, a pX330 plasmid encoding the target sequence of *SLC35B2* (5′-TCCGCCTGAAGTACTGCACC-3′) or a pX330-EGFP plasmid encoding the target sequence of *B3GAT3* (5'- GTTCCGCTGCTCGACACCAC-3′; a gift from Dr. Maeda) was transfected into HEK293T cells, respectively. Anti-HS mAb- and GFP-negative single clones were then obtained using a cell sorter (SH800, Sony). B3GAT3-transfected *B3GAT3* KO or SLC35B2-transfected *SLC35B2* KO HEK293T cells were generated by stable transfection of B3GAT3 or SLC35B2 into *B3GAT3* KO or *SLC35B2* KO HEK293T cells, respectively, using the pMxs-puro retroviral expression system as described above.

### Preparation of SARS-CoV-2 spike pseudovirus and titration

HEK293T cells were transiently transfected with the plasmids for the SARS-CoV-2 spike protein of D614G, BA.1, BA.2, and BA.4/5 using Lipofectamine LTX. Twenty-four hours after transfection, VSV/ΔG-Luc pseudoviruses carrying the VSV-G protein were inoculated in DMEM without FBS for 2 hours at 37°C. The cells were then carefully washed with FBS-free DMEM, and the medium was replaced with DMEM containing 10% FBS and 1% PS. After incubation for 24 hours at 37°C, the supernatants containing the SARS-CoV-2 spike pseudoviruses were harvested and centrifuged at 470 × *g* for 2 min at 4°C and at 1,300 × *g* for 2 min at 4°C. Aliquots of the pseudovirus were stored at −80°C until use. To determine pseudovirus titers, VSV-G transfectants were mixed with serially diluted pseudovirus for 18 hours at 37°C in a 96-well plate. Luciferase activity was measured by a ONE-Glo Luciferase Assay System (Promega, E6120) using a luminescence plate reader (Tristar LB94, Berthold Technologies). Normalized luminescence values were calculated using min-max normalization.

### SARS-CoV-2 spike pseudovirus entry assay

Unless otherwise indicated, cells were seeded in collagen (Cellmatrix Type I-C, Nitta Gelatin)-coated 96-well plate at 40,000 cells per well 1 day before infection and were infected with SARS-CoV-2 spike pseudovirus in DMEM without FBS for 1 hour at 37°C with agitation. Thereafter, infected cells were cultured for 18 hours at 37°C with DMEM containing 10% FBS and 1% PS, and luciferase activity was analyzed. For the heparinase treatment, cells were pretreated with Heparinase I and III (2 U/mL) in DMEM without FBS for 30 min at 37°C before infection. For the heparin inhibition assay, the pseudovirus was premixed with heparin at the indicated concentrations before infection. Dose-response curves for the inhibition of pseudovirus infection in HEK293T cells by heparin were calculated using a four-parameter non-linear regression model with GraphPad Prism version 7.0e.

### Authentic SARS-CoV-2 virus infection assay

Cells were seeded at 40,000 cells per well in a collagen-coated 96-well plate 1 day before infection and were then infected with authentic SARS-CoV-2 virus (8,000 TCID_50_) in DMEM without FBS for 1 hour at 37°C with agitation. After infection, the medium was replaced with DMEM containing 10% FBS and 1% PS, infected cells were incubated for 24 hours at 37°C, and then the cell culture supernatants or cell lysates were analyzed by reverse transcription-quantitative polymerase chain reaction (RT-qPCR). For the heparin inhibition assay, the virus was premixed with heparin at the indicated concentrations prior to infection.

### RT-qPCR analysis of SARS-CoV-2 infection

Supernatants from infected cells were centrifuged at 500 × *g* for 5 min. The resulting supernatants were either treated with 2% proteinase K (Takara, 9034) for 15 min at 55°C, followed by inactivation for 5 min at 98°C, or subjected to viral RNA extraction using QIAamp Viral RNA Mini Kits (Qiagen, 52906). For analysis of cell lysates, cells were lysed with RLT buffer (Qiagen, included in RNeasy Mini Kit, 74104), and viral RNA was extracted using an RNeasy Mini Kit (Qiagen, 74104). RT-qPCR was performed using a One Step TB Green PrimeScript RT-PCR Kit II (Takara, RR086B) with the QuantStudio 3 Real-Time PCR System (Thermo Scientific). SARS-CoV-2 RNA was used to generate a standard curve. The following primers were used: for SARS-CoV-2 N; 5'- AGCCTCTTCTCGTTCCTCATCAC-3′ and 5'- CCGCCATTGCCAGCCATTC −3', and for *Actb*; 5'- GCGAGAAGATGACCCAGATC-3′ and 5'- GGATAGCACAGCCTGGATAG −3'.

### Infection of human nasal epithelial cells

Primary human nasal epithelial cells (MucilAir EP02MP, Epithelix Sàrl) were purchased and handled according to the manufacturer’s instructions. The cells were infected with authentic SARS-CoV-2 virus (20,000 TCID_50_) in DMEM without FBS at 37°C for 1 hour. After the apical surface of the cells was washed once with PBS, 20 min apical washes were collected at 1 and 24 hours post-inoculation (hpi). Cell lysates were harvested at 24 hpi. For the heparin inhibition assay, the virus was premixed with 100 µg/mL heparin prior to infection.

### CRISPR KO library screening

CRISPR KO library-transduced HEK293T cells were generated by lentivirus-mediated transduction using the Human CRISPR KO Pooled Library (GeCKO v2; Addgene, 1000000048). Briefly, the lentiCRISPRv2 plasmid for the sgRNA library, pantropic envelope VSV-G, and HIV packaging genes (gag-pol, rev) were co-transfected into HEK293T cells. The cell culture supernatants containing the lentiviruses were harvested 2 days later, and HEK293T cells were infected with the lentivirus library at 1,200 × *g* for 2 hours at 32°C. CRISPR KO library-transduced HEK293T cells were treated with 3 µg/mL puromycin for 3 days, and then BA.1 N417K RBD-Fc (30 µg/mL) non-binding cells were enriched by 4× cell sorting (SH800, Sony). To verify that BA.1 RBD non-binding cells were obtained, the cells were stained with BA.1 RBD-Fc (10 µg/mL) before and after sorting. After screening, genomic DNA was harvested using a WIZARD SV Genomic DNA Purification System (Promega, A2361), and sgRNA sequences were amplified by PCR and cloned into the plasmid. sgRNA sequences derived from the library were analyzed by a DNA sequencer (ABI3130xl).

### Electrostatic potential surfaces of proteins

The electrostatic potential surface of RBD (WT, PDB 6M0J; BA.1, PDB 7WBP; BA.2, PDB 7XB0; BA.4/5, PDB 7XWA; XBB.1, PDB 8IOV; BA.2.86, PDB 8QSQ) was calculated using the Adaptive Poisson-Boltzmann Solver algorithm (70) and visualized using PyMOL version 2.5.2.

### Docking simulation analysis

The docking server ClusPro (https://cluspro.org) was used for identifying a potential heparin-binding site (32–36). The server-generated 10 poses of tetrasaccharide heparin docked to RBD.

### Biotinylation of HSPG

Heparan sulfate proteoglycan (Sigma-Aldrich, H4777) was biotinylated with EZ-Link Sulfo-NHS-LC-Biotin (Thermo Scientific, 21335), followed by buffer exchange with Zeba Spin Desalting columns (Thermo Scientific, 89883).

### HSPG/ACE2 binding assay

Flag-tagged spike or Flag-tagged RBD-TM and GFP were co-transfected into *B3GAT3* KO HEK293T cells using Lipofectamine LTX. After 48 hours of transfection, transfected cells were incubated with biotinylated HSPG (2 µg/mL), biotinylated ACE2-Fc protein (2 µg/mL), or anti-Flag mAb (0.017 µg/mL) for 30 min at 4°C, followed by detection of the HSPG or anti-Flag mAb bound spike or RBD protein using APC-conjugated streptavidin (1.3 µg/mL) or APC-conjugated anti-rat IgG (H + L) Ab (2.5 µg/mL). Dead cells were stained with PI. HSPG or ACE2 binding on GFP-expressing cells in the live cells was analyzed using flow cytometers (FACSVerse, BD Biosciences or Attune, Thermo Scientific). To evaluate HSPG or ACE2 binding, GMFI values were measured for both GFP-positive and GFP-negative cells, and the difference was calculated. Expression levels of the spike or RBD protein were adjusted by anti-Flag mAb staining and normalized to the value of BA.1. The gating strategy is shown in Fig. S1.

### HSPG/ACE2 binding inhibition assay by anti-RBD mAbs

Flag-tagged BA.1 spike protein and GFP co-transfected *B3GAT3* KO HEK293T cells were mixed with various concentrations of anti-RBD monoclonal Abs for 30 min at 4°C, followed by incubation with biotinylated HSPG or biotinylated ACE2-Fc protein at 2 µg/mL for 30 min at 4°C. Thereafter, the HSPG or ACE2 bound to the BA.1 spike protein was detected using APC-conjugated streptavidin (1.3 µg/mL). Dead cells were stained with PI. The stained cells in the live cells were then analyzed using a flow cytometer (FACSVerse, BD biosciences). Dose-response curves for the inhibition of HSPG or ACE2 binding to the BA.1 spike protein by mAbs were calculated using a four-parameter non-linear regression model with GraphPad Prism version 7.0e.

### Immunofluorescence stain

Formalin-fixed, paraffin-embedded tissue sections of nasal tissue were purchased from OriGene. This tissue section was stained with anti-HS mAb (14 µg/mL), followed by Alexa Fluor 555-conjugated anti-mouse IgM Ab (5.0 µg/mL). Nuclei were counterstained with DAPI. The stained tissue section was analyzed using an IX81 fluorescence microscope (Olympus).

### Flow cytometric analysis and SARS-CoV-2 spike pseudovirus or authentic SARS-CoV-2 virus infection assay for TMPRSS2 expressing cells

TMPRSS2 and GFP were co-transfected into HEK293T cells or ACE2-stably expressing HEK293T cells using Lipofectamine LTX. The ratio of the plasmid DNA amount of TMPRSS2 to that of GFP is 0.33:1 or 1:1. After 48 hours of transfection, transfectants were stained with WT or BA.1 RBD-Fc (10 µg/mL), mouse anti-syndecan-1 mAb (5.0 µg/mL), mouse anti-glypican-4 mAb (5.0 µg/mL), mouse anti-HS mAb (5.0 µg/mL), mouse anti-ACE2 mAb (5.0 µg/mL), mouse anti-CD46 mAb (2.0 µg/mL), mouse anti-CD59 mAb (5.0 µg/mL), mouse anti-integrin αV mAb (2.0 µg/mL), or mouse anti-HLA class I mAb (3.3 µg/mL) for 30 min at 4°C. Thereafter, RBD-Fc- or mAb-bound cells were detected using APC-conjugated anti-human IgG Fc Ab (1.3 µg/mL), APC-conjugated anti-mouse IgG Fc Ab (1.3 µg/mL), or APC-conjugated anti-mouse IgM Ab (2.5 µg/mL). Dead cells were stained with PI. Antibody binding on GFP-expressing cells in the live cells was analyzed by flow cytometer (FACSVerse, BD Biosciences or Attune, Thermo Scientific). GFP-positive cells among the TMPRSS2 and GFP co-transfected cells were purified by cell sorter (MA900, Sony). Sorted cells (40,000 cells per well) were cultured for 1 day in a collagen-coated 96-well plate. Thereafter, SARS-CoV-2 spike pseudovirus or authentic SARS-CoV-2 virus infection assay was performed.

### Data and statistical analysis

FlowJo version 10.9 (BD Biosciences) was used to analyze the flow cytometry data, and GraphPad Prism version 7.0e was used for graph generation and statistical analysis.

## Data Availability

The data supporting the findings of this study are available from the corresponding author on request.

## SUPPLEMENTAL MATERIAL

Fig. S1 mbio.01303-25-s0001.pdf Gating strategy for HSPG or ACE2 binding assay.

ASM does not own the copyrights to Supplemental Material that may be linked to, or accessed through, an article. The authors have granted ASM a non-exclusive, world-wide license to publish the Supplemental Material files. Please contact the corresponding author directly for reuse.
