# Supplementary material for: Evolutionary dynamics of heparan sulfate utilization by SARS-CoV-2
Source: mBio. 2025 Jun 23;16(8):e01303-25. doi: 10.1128/mbio.01303-25 (PMC12345153; doi:10.1128/mbio.01303-25)
Supplement: Fig. S1 — Gating strategy for HSPG or ACE2 binding assay. [file mbio.01303-25-s0001.pdf]

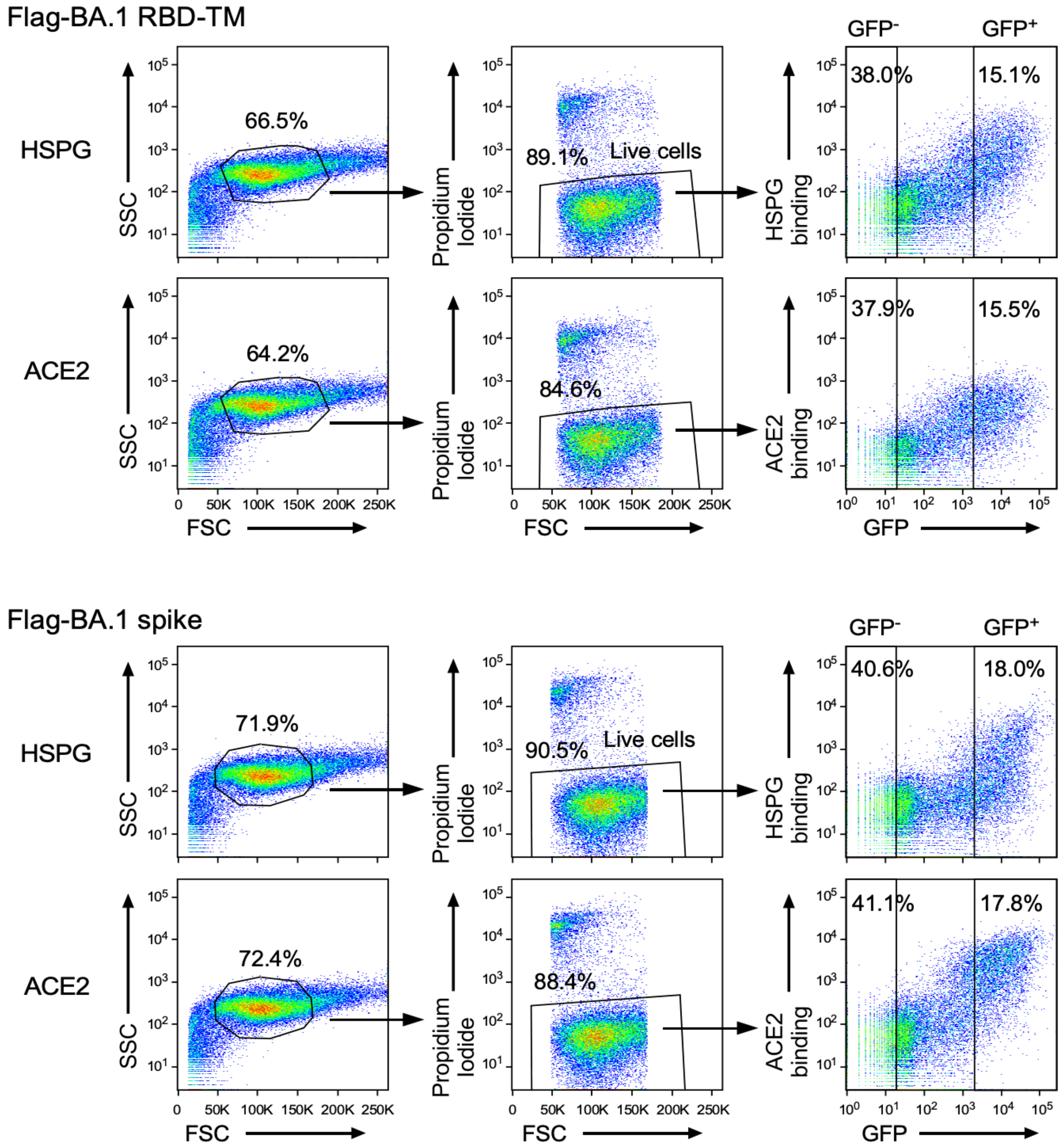

Figure S1. Gating strategy for HSPG or ACE2 binding assay. Representative gating strategy. *B3GAT3* KO HEK293T cells co-transfected with GFP and either Flag-tagged BA.1 RBD-TM (upper) or Flag-tagged BA.1 spike protein (lower) were first gated based on FSC and SSC. Dead cells were excluded by gating out propidium iodide-positive cells. HSPG or ACE2 binding in live cells was quantified by comparing the GMFI of GFP-positive (GFP^+) and GFP-negative (GFP^-) populations.
